# Plasma Vascular Endothelial Growth Factor Concentration and Alveolar Nitric Oxide as Potential Predictors of Disease Progression and Mortality in Idiopathic Pulmonary Fibrosis

**DOI:** 10.3390/jcm5090080

**Published:** 2016-09-07

**Authors:** Jalpa Kotecha, Ludmila Shulgina, Darren W. Sexton, Christopher P. Atkins, Andrew M. Wilson

**Affiliations:** Norfolk and Norwich University Hospital, Colney Lane, Norwich NR4 7UY, Norfolk, UK; ludmila.shulgina@nhs.net (L.S.); D.W.Sexton@ljmu.ac.uk (D.W.S.); c.atkins@uea.ac.uk (C.P.A.); a.m.wilson@uea.ac.uk (A.M.W.)

**Keywords:** idiopathic pulmonary fibrosis, vascular endothelial growth factor, alveolar nitric oxide

## Abstract

**Background**: Declining lung function signifies disease progression in idiopathic pulmonary fibrosis (IPF). Vascular endothelial growth factor (VEGF) concentration is associated with declining lung function in 6 and 12-month studies. Alveolar nitric oxide concentration (CANO) is increased in patients with IPF, however its significance is unclear. This study investigated whether baseline plasma VEGF concentration and CANO are associated with disease progression or mortality in IPF. **Methods**: 27 IPF patients were studied (maximum follow-up 65 months). Baseline plasma VEGF concentration, CANO and pulmonary function tests (PFTs) were measured. PFTs were performed the preceding year and subsequent PFTs and data regarding mortality were collected. Disease progression was defined as one of: death, relative decrease of ≥10% in baseline forced vital capacity (FVC) % predicted, or relative decrease of ≥15% in baseline single breath diffusion capacity of carbon monoxide (TLCO-SB) % predicted. **Results**: Plasma VEGF concentration was not associated with progression-free survival or mortality. There was a trend towards shorter time to disease progression and death with higher CANO. CANO was significantly higher in patients with previous declining versus stable lung function. **Conclusion**: The role of VEGF in IPF remains uncertain. It may be of value to further investigate CANO in IPF.

## 1. Introduction

Idiopathic pulmonary fibrosis (IPF) is a progressive disease characterised by irreversible distortion of the lung architecture [1] with histological changes including honeycombing, sparse cellular inflammation and fibroblastic foci [2]. It carries a poor prognosis, with a mean survival of 2.9 years from the time of diagnosis [3,4], however, there is considerable variability in outcome and prognostic indicators are required. Other than a change in lung function [5,6,7,8,9,10,11], no prognostic modelling tools are routinely used in clinical practice.

Several studies have highlighted the importance of angiogenesis in the pathophysiology of IPF [12,13,14] and fibroblastic foci exhibit substantially decreased vascular density, whereas adjacent non-fibrotic tissue is highly vascularised [15,16,17,18,19]. Vascular endothelial growth factor (VEGF) is a glycoprotein pluripotent growth factor and a fundamental regulator of normal and pathological angiogenesis [20,21]. VEGF expression has been found to be increased in capillary endothelial cells and type II alveolar epithelial cells in highly vascularised alveolar septa and areas of lung spared of fibrotic changes, with the converse true in severely fibrotic areas [15,22]. Nintedanib, which inhibits the VEGF receptor-2 (VEGFR-2), was shown to specifically reverse VEGF-induced proliferation in human primary lung fibroblasts from IPF patients [23,24].

Previous studies have yielded conflicting results regarding the relationship between serum or plasma VEGF concentration and prognosis in IPF. Ando et al. found a significant inverse correlation between baseline serum VEGF concentration and the change per month in vital capacity over 12 months, and a tendency towards shorter survival with higher baseline VEGF concentrations in a study of 41 patients with IPF [1]. Simler et al., in a study of 49 patients with interstitial lung disease (ILD) (39 of whom had IPF), found a significant negative relationship between change in plasma VEGF concentration and change in % predicted forced vital capacity (FVC) over 6 months [3]. By contrast, Ventetuolo et al. [25], in a cross-sectional analysis of 52 IPF patients, failed to find any association between plasma VEGF concentration and pulmonary arterial pressure, pulmonary vascular resistance, FVC, diffusion capacity of carbon monoxide (DLCO) or functional measures such as the 6 min walk test distance (6MWD) or New York Heart Association (NYHA) class. The role of VEGF in the pathophysiology and prognosis of IPF is therefore unclear.

The concentration of airway nitric oxide (NO) has been used as a marker of inflammation in obstructive lung diseases such as asthma, however its role in IPF is poorly clarified [26]. Vyas-Read et al. [27] hypothesised that chronic elevation of profibrotic cytokines leads to a downregulation of nitric oxide synthase (NOS), resulting in the transition of alveolar epithelial cells to the myofibroblast phenotype and consequent pulmonary fibrosis. NO concentration in breath exhaled at 50 mL/s (FENO50) as well as the alveolar concentration of NO (CANO) have been shown to be significantly higher in IPF patients compared to both healthy controls and sarcoidosis patients [28]. In another fibrotic lung disease, systemic sclerosis (SSc), studies have shown that CANO is significantly higher in patients with SSc compared to healthy controls and that CANO levels are significantly higher in SSc patients with ILD compared to those without ILD [29]. CANO has therefore been proposed as a useful marker for making a diagnosis of and monitoring progression of ILD in SSc [29,30,31,32]. Further information is required to determine if CANO can be used in a similar role for IPF.

Given the desire to determine better methods of predicting prognosis, our study aimed to explore whether plasma VEGF concentration or CANO are associated with progression-free survival or mortality in IPF over a 5 year period.

## 2. Methods

This study was conducted in accordance with Good Clinical Practice including Research Ethics Committee (09/H0310/78) and all participants gave written informed consent. Patients were consented between March and June 2010.

### 2.1. Patients

Patients were identified at the respiratory clinic at the Norfolk and Norwich University Hospital, Norwich. Eligible patients had a clinically labelled diagnosis of IPF with High Resolution Computerised Tomography (HRCT) scan features compatible with Usual Interstitial Pneumonia (UIP) or histological features, in keeping with a diagnosis of IPF as per current guidelines [33], and were able to provide informed consent. Histology was not required as an entry criterion. Patients were greater than 40 years old; none had an exacerbation of their disease, respiratory tract infection or changes in their immunosuppression treatment within 6 weeks of recruitment.

Patients were excluded from the study if a secondary cause for pulmonary fibrosis was identified, if they had a recognised significant co-existing respiratory disorder or had a significant medical, surgical or psychiatric disease that would affect subject safety or influence the study outcome. Other exclusion criteria included current smokers and current pregnancy or breast-feeding.

### 2.2. Study Design

Patients attended the clinic for a screening visit and baseline measurements including history, medical examination, and assessment of alveolar nitric oxide. Venepuncture was performed to obtain peripheral venous blood for plasma VEGF analysis, following informed consent. Lung function data was obtained from 6 to 12 months prior to recruitment to the study and patients were defined as stable or decliners (decline in lung function being defined as one or both of: ≥10% relative decline in FVC % predicted or ≥15% relative decline in single breath diffusion capacity of carbon monoxide (TLCO-SB) % predicted). All other data was obtained from the medical notes. Data from subsequent routine clinic spirometry was recorded for a period of up to 65 months; the frequency of follow-up visits varied between patients.

Subsequent data regarding mortality was collected using the Sunquest ICE (Integrated Clinical Environment) system at the Norfolk and Norwich University Hospital.

### 2.3. Exhaled Nitric Oxide Measurement

Exhaled nitric oxide was measured using a NIOX nitric oxide analyzer at baseline visit (Aerocrine, Chicago, IL, USA), with an expiratory flow rate of 50 mL/s (FENO-50) according to American Thoracic Society guidelines [33], as well as at 30, 100 and 200 mL/s. An attempt to obtain three valid FENO measurements was undertaken at each of the four flow rates for each patient. The mean of three separate measures of nitric oxide at each flow rate was used in the analysis. A model by Tsoukias and George was used to estimate CANO (in units of ppb) [34,35,36]. The analyser was calibrated fortnightly using a cylinder of nitric oxide at a concentration of 208 ppb.

### 2.4. Vascular Endothelial Growth Factor (VEGF)

Venous blood was centrifuged within 1 h of collection at 300 g for 10 min. Plasma was separated and stored at −80 °C. All samples were analysed simultaneously after being kept in a freezer for 18–24 months. The concentration of VEGF in plasma was assessed using a commercially available validated Human VEGF-A Platinum Enzyme-linked immunosorbent assay (ELISA) kit (eBioscience, Bender MedSystems GmbH, Vienna, Austria). According to a manufacturer’s brochure, an expected VEGF-A concentration in plasma of healthy donors was estimated as a mean value of 45.7 pg/mL.

### 2.5. Measurement of Progression

Progression in lung disease was defined as one or more of: death, a relative decrease of ≥10% in baseline FVC % predicted, or a relative decrease of ≥15% in baseline TLCO-SB % predicted. Time to progression was defined as the number of months until the occurrence of the earliest of one of the three events defining progression.

### 2.6. Statistical Analysis

Two-tailed *t*-tests were performed to assess for any difference in mean baseline VEGF concentration and CANO according to mortality status, age (older versus younger than the group’s median age), gender, use of immunosuppressants, use of Long Term Oxygen Therapy (LTOT), previous stable versus declining lung function and the presence of concurrent emphysema (taken from diagnosis lists on clinic letters). Two-tailed *t*-tests were also performed to assess for any difference in mean time to disease progression or death for patients with previous stable versus declining lung function. The Spearman’s rank correlation coefficient was calculated to assess for any correlation between decline in lung function and time to death.

Kaplan-Meier survival analysis was performed to assess whether plasma VEGF concentration, CANO or previous stable or declining lung function were associated with either time to disease progression or death. Patients were split into groups according to low (less than the whole group median) or high (greater than the whole group median) plasma VEGF concentration and CANO.

Cox regression analysis was used to determine hazard ratios for mortality and disease progression according to baseline plasma VEGF concentration and CANO, again after splitting patients into two groups for each of the parameters as described for the Kaplan-Meier analysis.

For all tests, a *p*-value of <0.05 was considered to be significant.

All statistical analysis was performed using IBM SPSS version 22.

## 3. Results

Thirty-one patients were initially recruited (25 males, six females). Three patients were excluded due to inadequate evidence for a diagnosis of IPF (as defined above) and one patient was lost to follow-up. Of the remaining 27 patients, there were 23 males and four females. The median age was 73.0 years. Seven patients had concurrent emphysema reported on their Computerised Tomography (CT) scan but no clinical or pulmonary function evidence of airway obstruction. Five patients were taking immunosuppressants (three receiving prednisolone only, two receiving prednisolone and azathioprine) and two were receiving LTOT. Sixteen patients had previous stable lung function. VEGF concentration was obtained for 26 patients, and measurements of CANO for all patients. The median follow-up after measurement of VEGF concentration and CANO was 33 months (standard deviation = 20.4 months, range = 1–65 months). Table 1 and Table 2 summarise the baseline demographics, lung function and measurements of plasma VEGF concentration and CANO.

Over the course of 5 years, 21 patients died; all patients met the criteria for disease progression as outlined above. The median time to death was 33 months, whereas the median time to progression was 13 months.

The frequency of subsequent pulmonary function tests was as follows: all 26 patients who were alive at 6 months had had a further pulmonary function test, 21 had had at least one further test between 6 and 12 months (including one patient who died between 6 and 12 months), and 18 (of 23 patients alive at 18 months) had had at least one further test between 12 and 18 months.

A significant difference in mean CANO was found between patients having previous stable compared to declining lung function (mean CANO 3.84 ppb for patients with previous stable lung function versus 6.05 ppb for those with previous declining lung function, *t* = −2.48, *p* = 0.02). No significant difference in mean CANO was found according to gender, age, use of immunosupressants or LTOT, presence of concurrent emphysema or mortality status. No significant difference was found in mean plasma VEGF concentration for any of the variables above. No significant difference was found in the mean time to disease progression or death when comparing patients with previous stable versus declining lung function.

Total time in months of follow-up (used as a marker of survival time; either time to death or time to the end of the study) was significantly positively correlated with time in months to reach a relative decline in FVC % predicted of ≥10% (*r* = 0.762, *p* < 0.001).

**Figure 1:**
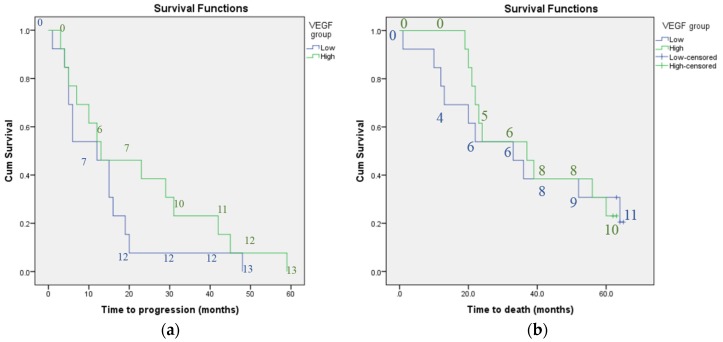
Kaplan-meier curves: survival in terms of: (**a**) disease progression and (**b**) mortality according to baseline plasma VEGF concentration; Kaplan-meier curves showing no significant difference in survival as measured by progression of lung disease or mortality according to baseline plasma VEGF concentration; Key: low = baseline plasma VEGF concentration less than group median; high = baseline plasma VEGF concentration greater than group median.

**Figure 2:**
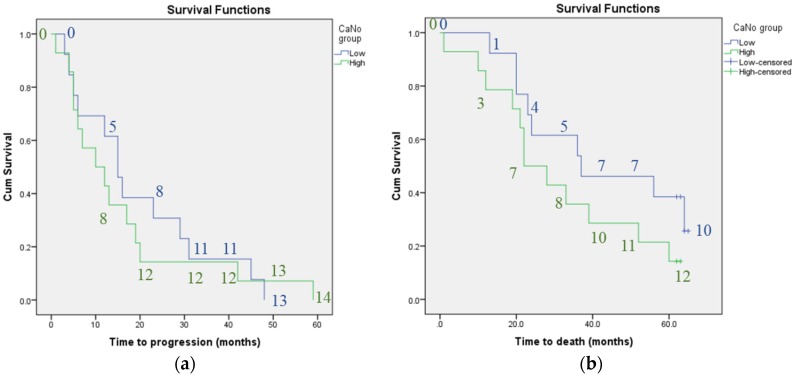
Kaplan-meier curves: survival in terms of: (**a**) disease progression and (**b**) mortality according to baseline CANO; Kaplan-meier curves showing a non-significant trend towards shorter survival as measured by progression of lung disease or mortality in patients with high versus low baseline CANO; Key: low = baseline CANO less than group median; high = baseline CANO greater than group median.

**Figure 3:**
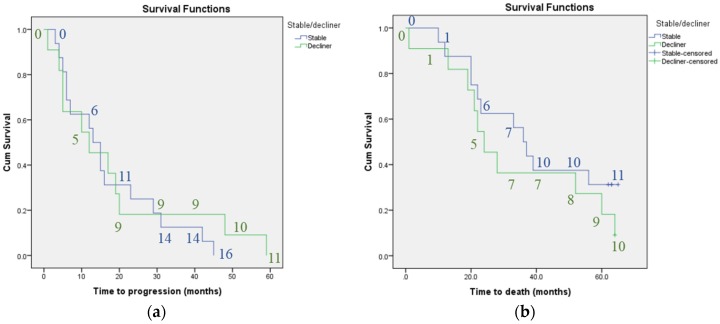
Kaplan-meier curves: survival in terms of: (**a**) disease progression and (**b**) mortality according to previous stable versus declining lung function; Kaplan-meier curves showing a non-significant trend towards shorter survival as measured by progression of lung disease or mortality in patients with previous declining versus stable lung function (over 6 or 12 months prior to recruitment to study).

Kaplan-Meier analysis did not find plasma VEGF concentration or CANO to be associated with progression-free survival or mortality. Whilst there was a trend towards shorter survival time (median survival time 22 months for patients with high CANO compared to 37 months for those with low CANO) and shorter time to disease progression (median time 10 compared to 15 months for patients with high versus low CANO) with higher CANO, the confidence intervals overlapped. There was also a trend towards shorter survival time and shorter time to disease progression for patients with previous declining versus stable lung function, however, again the confidence intervals overlapped (median survival time 24 compared to 36 months and median time to progression 12 versus 13 months for patients with previous declining versus stable lung function). Figure 1, Figure 2 and Figure 3 illustrate the Kaplan-Meier survival curves for baseline plasma VEGF concentration, CANO and previous trend in lung function respectively.

No. of patients in low VEGF group = 13; no. of patients in high VEGF group = 13. The numbers on the graphs indicate the number of individuals classified as having progressive disease or who had died at each 10-month interval for each subgroup.

No. of patients in low CANO group = 13; no. of patients in high CANO group = 14. The numbers on the graphs indicate the number of individuals classified as having progressive disease or who had died at each 10-month interval for each subgroup.

No. of patients with previous stable lung function = 16; no. of patients with previous declining lung function = 11. The numbers in the graphs indicate the number of individuals classified as having progressive disease or who had died at each 10-month interval for each subgroup.

Cox regression analysis did not show any significant results when looking at plasma VEGF concentration or CANO (Table 3). Therefore, although there was a trend towards shorter survival in patients with a higher baseline CANO, the hazard ratio was not significantly increased in this patient group.

## 4. Discussion

Our study failed to identify a significant association between plasma VEGF concentration or CANO and time to disease progression or death. Neither plasma VEGF concentration nor CANO were predictive of survival. However, we did find that mean CANO was significantly higher in patients with previous declining versus stable lung function. There was also a trend towards shorter survival time and shorter time to disease progression in patients with high CANO, suggesting that this may be a useful parameter to study in the future. The fact that the trends regarding survival time and disease progression were observed over a 5 year time period adds weight to the argument for further studies in this area given that the mean survival time of IPF patients from diagnosis is 2.9 years.

Experimental studies have shown conflicting evidence regarding the effect of VEGF on pulmonary fibrosis, reflecting the uncertainty surrounding its impact on the disease process. Some studies have suggested that deleting the VEGF gene or inhibiting it leads to attenuation of pulmonary fibrosis [22,37,38] whereas others have shown the opposite effect [39,40]. Stockmann et al. suggested that the discrepancy observed between various studies in the effect of VEGF inhibition on pulmonary fibrosis could be due to the fact that, although VEGF inhibitors may inhibit early damage, VEGF may be important for subsequent repair processes and so inhibiting VEGF in this situation would in fact be detrimental [40]. Whilst previous studies have suggested that VEGF concentration is related to decline in lung function [1,3], our study did not find VEGF concentration to be predictive of disease progression as measured by decline in lung function over a 5 year period. The fact that the results of our study are not in keeping with previous findings adds to the uncertainty surrounding the role of VEGF in IPF.

In the study performed by Simler et al. [3], correlation was elicited between a 6-month change in plasma VEGF concentration and a 6-month change in % predicted FVC as opposed to comparing baseline VEGF concentration with subsequent change in % predicted FVC. Change in VEGF concentration may therefore be a better indicator of change in lung function than baseline VEGF concentration. Their baseline analysis only elicited a significant negative correlation between baseline plasma concentration of endothelin-1 and % predicted FVC and plasma concentration of interleukin-8 and % predicted TLCO; no significant correlations at baseline were reported between plasma VEGF concentration and lung function parameters.

The analysis performed by Ando et al. over a 12-month period [1] looked at serum as opposed to plasma VEGF concentration. Due to the lack of studies in this area (existing studies have involved cancer patients or healthy volunteers) it is unclear whether plasma or serum VEGF is the better measure in IPF specifically. Serum concentration of VEGF has been shown to be affected by the production of VEGF by platelets and it has been suggested to be a less suitable measure of VEGF concentration than plasma VEGF, although there has been some suggestion for its utility in cancer patients where platelet-derived pathology is thought to reflect the biology of cancer cells [41,42,43] . Platelet reactivity has been shown to be increased in IPF [44], however, the significance of this is, as yet, unclear.

We showed a significant positive correlation between time to death and time taken to reach a ≥10% relative decline in FVC % predicted. This finding is in keeping with previous study results, which have led to a decline in lung function being used as a marker for disease progression. This implies that our lung function data was robust, and makes it less likely that the lack of association observed between VEGF concentration and CANO and change in lung function was due to inaccuracies in the measurements of lung function.

Whilst disease progression, as defined by declining lung function, was associated with time to death as discussed above, there was a non-significant trend towards shorter time to disease progression and earlier death with previous declining versus stable lung function. Whilst decline in lung function has been shown to be a good predictor of mortality in populations [6,7,8,9,10], there is emerging evidence that previous decline in lung function is not a good predictor of subsequent decline in lung function on an individual patient basis due to a substantial amount of intra-subject variability in measurements [45]. In a study by Schmidt et al., decline in lung function was shown to predict mortality but not future decline in lung function [46].

Our study had several limitations. The mean time between diagnosis of IPF and recruitment to our study was 35 months. In order to more accurately establish a baseline and evaluate the relationship between disease-free progression and mortality with VEGF and CANO levels, it would have been ideal to recruit patients with incident diagnoses of IPF. Whilst studying incident cases is, in itself, not free from bias due to differences in lead-in time, it would likely have led to a more accurate assessment of any relationship between the parameters studied. The study population included seven patients with concurrent emphysema. These patients were included in our study as the emphysema was not thought to be a significant contributing factor to their respiratory function. Whilst there is evidence that patients with IPF and concurrent emphysema are of a different phenotype than those with IPF alone, the decision to include these patients was a pragmatic one as we felt this reflected what would be encountered in clinical practice. Many patients with IPF are ex-smokers and are therefore likely to have a degree of concurrent emphysema. Nonetheless, the majority of participants recruited did not have concurrent emphysema.

Two patients had previously been exposed to prednisolone and azathioprine. Recruitment to our study was carried out before publication of the PANTHER-IPF trial and the finding of increased morbidity and mortality in patients receiving these drugs [47]. It is possible that the two patients who received combination therapy with prednisolone and azathioprine may have suffered adverse effects due to this, affecting their disease course. As the results of the PANTHER-IPF trial were not available at the time of recruitment, this was not something we could have considered when determining our exclusion criteria.

The patients had lung function follow-up at varying time intervals, and the lack of standardisation of frequency of lung function tests meant that it was difficult to accurately compare the time at which a patient was defined as having progressive lung disease. The number of patients in our study was small compared to previous studies looking at VEGF concentration in IPF patients [1,3]. This meant that it was difficult to compare our results to those of other groups in terms of reliability. However, we did have a long duration of follow-up, enabling assessment of the relationship of VEGF with lung function over a longer period of time than in previous studies. Lastly, we did not have control patients in our study. It would have been useful to compare CANO values in our patient group with those of healthy controls to potentially add weight to the case for future research to assess whether CANO is a useful marker in IPF.

## 5. Conclusions

Our study did not find any association between plasma VEGF concentration measured at baseline and mortality or progression-free survival in IPF. This contradicts findings of previous studies and reflects the uncertainty regarding the role of VEGF in IPF. It is unclear what effect measuring serum versus plasma VEGF concentration has on findings, and whether it is purely the change in plasma VEGF concentration that is predictive of subsequent decline, or whether baseline VEGF concentration also has value in this role. Future studies of larger patient cohorts may clarify these points.

The mean CANO was significantly higher in patients with declining lung function compared to those with stable lung function over the year preceding recruitment to the study. There was a trend towards earlier mortality and a shorter duration of progression-free survival in patients with high versus low CANO. This suggests that it may be useful to further investigate CANO as a possible predictor of decline in IPF in a larger study.

## Figures and Tables

**Table 1:** Summary table of patient demographics and baseline lung function.

Demographic	Number of Patients or Mean Parameter Values
Male sex (*n* (%))	23 (85%)
Ex-smokers * (*n* (%))	19 (70%)
Receiving LTOT at recruitment (*n* (%))	2 (7%)
Receiving immunosuppressants at recruitment (*n* (%))	5 (19%)
Age (years) (mean (S.D.))	72.8 (9.5)
Disease duration (months) (mean (S.D.))	35.0 (27.0)
Baseline FVC % predicted (mean (S.D.))	71.8 (18.1)
Baseline TLCO-SB % predicted (mean (S.D.))	43.3 (16.0)

Key: *n* = number of patients; % = percentage of whole group; S.D. = standard deviation; Disease duration describes the length in time between diagnosis of IPF and recruitment to the study; * There were no current smokers in the study.

**Table 2:** Summary table of baseline plasma VEGF concentration and CANO.

Parameter	Subgroup	Median	Range
Plasma VEGF concentration (pg/mL)	Combined	133.0	286.4
	High	169.2	202.6
	Low	82.5	71.1
C_A_NO (ppb)	Combined	4.4	10.5
	High	6.4	6.6
	Low	3.0	3.9

Key: Combined = data for whole group; high = data for subgroup with values greater than the whole group median; low = data for subgroup with values less than the whole group median.

**Table 3:** Cox regression analysis: prediction of mortality or disease progression using baseline plasma VEGF concentration and CANO.

Parameter	Time to Death (Months)			Time to Progression (Months)		
	Hazard Ratio	95% CI Lower Bound	95% CI Upper Bound	Hazard Ratio	95% CI Lower Bound	95% CI Upper Bound
Plasma VEGF concentration	0.93	0.38	2.28	0.62	0.28	1.40
C_A_NO	1.88	0.77	4.62	0.96	0.84	1.10

Cox regression analysis showing no difference in the hazard ratios for time to death or progression of lung disease according to baseline plasma VEGF concentration or CANO. Key: 95%, CI = 95% confidence interval.
